# Role of Polymeric Excipients in the Stabilization of Olanzapine when Exposed to Aqueous Environments

**DOI:** 10.3390/molecules201219832

**Published:** 2015-12-14

**Authors:** Maria Paisana, Martin Wahl, João Pinto

**Affiliations:** 1 iMed.ULisboa, Departamento de Farmácia Galénica e Tecnologia Farmacêutica, Faculdade de Farmácia, Universidade de Lisboa, Av. Prof. Gama Pinto, P-1649-003 Lisboa, Portugal; mdcpaisana@gmail.com; 2 Institut für Pharmazeutische Technologie und Biopharmazie, Pharmazeutisches Institut, Eberhard Karls Universität Tübingen, Auf der Morgenstelle 8, D-72076 Tübingen, Germany; Martin.Wahl@uni-tuebingen.de

**Keywords:** anhydrous, extrusion, hydrate, hydroxypropylcellulose, olanzapine, pellet, polyethyleneglycol, polymorphism, polyvinylpyrrolidone, spheronisation

## Abstract

Hydrate formation is a phase transition which can occur during manufacturing processes involving water. This work considers the prevention of hydration of anhydrous olanzapine and hydrate conversions in the presence of water and polymers (polyethyleneglycol; hydroxypropylcellulose; polyvinylpyrrolidone) in forming pellets by wet extrusion and spheronisation. Anhydrous olanzapine was added to water with or without those polymers prior to extrusion with microcrystalline cellulose. Assessment of olanzapine conversion was made by XRP-Diffraction; FTIR spectroscopy; calorimetry (DSC) and microscopy (SEM for crystal size and shape). The addition of water converted the anhydrous form into dihydrate B and higher hydrate; whereas polyethyleneglycol promoted a selective hydrate conversion into the higher hydrate olanzapine form. Both polyvinylpyrrolidone and hydroxypropylcellulose prevented the hydrate transformations of the anhydrous drug; the latter even in the presence of hydrate seeds. This may be explained by the higher H-bond ability; higher network association and higher hydrophobicity of hydroxypropylcellulose by comparison with polyethyleneglycol and polyvinylpyrrolidone; which could contribute to its higher affinity to the crystal surfaces of the hydrate nuclei/initial crystals and promoting steric hindrance to the incorporation of other drug molecules into the crystal lattice; thus, preventing the crystal growth. The addition of microcrystalline cellulose needed for the pellets production (final product) did not eliminate the protector effect of both hydroxypropylcellulose and polyvinylpyrrolidone during pellets’ processing and dissolution evaluation.

## 1. Introduction

Active pharmaceutical ingredients (APIs) are exposed to water in many pharmaceutical processes, such as wet granulation or wet extrusion, which can lead to the conversion of the anhydrate form into the hydrate form during processing. The subsequent drying processes may induce the dehydration of the API, possibly resulting in a solid-state form different from the original [1]. Phase transformation of an API is of pharmaceutical interest because such transformations may change the physical and chemical properties of the drug with an impact on its dissolution and bioavailability [2]. The hydrate formation in wet masses takes place via solvent-mediated transformations (SMT) involving three different steps: (1) dissolution of the anhydrate phase in the solvent used for the wet massing of the powders; (2) nucleation of the hydrate species and (3) growth of the hydrate phase [3,4]. Inhibiting one of these stages could be advantageous to maintaining the original crystal form of the API and avoiding a product containing a mixture of solid-state forms. This inhibition can be accomplished by the use of selected excipients, thus avoiding hydrate and hydrate conversions of the API during processing.

The process of extrusion-spheronisation is a technique which includes various processing steps, such as blending of the dry mass, wet massing, extrusion of the moist mass, spheronisation of the extrudate and drying. Depending on the API and excipients used, solvent-mediated polymorphic transformations (SMT) may take place during the wetting step [5], which may or may not be reversible after the drying step. For example, it has been observed that both theophylline and nitrofurantoin can hydrate during the wet extrusion process, transforming into theophylline and nitrofurantoin monohydrates, respectively. However, anhydrous theophylline was shown to have better wetting properties and, thus, formed hydrates more easily. This demonstrates that each drug has different stabilities in aqueous environments requiring different conditions for hydration to occur [5,6]. During drying, theophylline monohydrate was shown to dehydrate easier and at relatively lower temperatures (60 °C) than nitrofurantoin monohydrate (135 °C). Therefore, inhibiting hydrate formation could be advantageous to maintaining the original crystal form by avoiding a product containing a mixture of solid-state forms, which may have an impact on the properties and stability of the API.

Microcrystalline cellulose (MCC) is extensively used in wet extrusion processes [7]. The effect of MCC on the reduction of the hydrate formation of the drugs during the wet massing step was shown to be originating from its ability to hold large amounts of water in its internal structure, which can retard the hydrate formation [2]. However, the use of high contents of water (e.g., 40%–60% *w*/*w*) may reduce the protection of MCC on retarding the hydrate formation. The addition of other excipients to the formulation (e.g., polymers) may reduce the amount of water needed for the powder to agglomerate. Furthermore, polymers are known to inhibit the nucleation and/or growth of hydrated pharmaceutical crystals in aqueous slurries [3,4]. For instance, polyvinylpyrrolidone (PVP) has been demonstrated to inhibit the growth rate of sulfathiazole and indomethacin in solutions [3]. The cross-linked poly(acrylic acid) (PAA) has been shown to completely inhibit the caffeine anhydrate to hydrate transformation in aqueous environments [4]. Cellulose derivatives (e.g., HPC and HPMC) have been shown to be successful in inhibiting the hydrate conversion of carbamazepine but exhibit no effect on the transformation kinetics of anhydrous sulfaguanidine [4]. The different inhibitory behaviors of different polymers, and the same polymer with different APIs, reveal that there is no single polymer which promotes the inhibition of hydrate conversions in all APIs. This may be a consequence of different specific interactions that the polymer may establish with individual crystal surfaces [8,9].

Olanzapine, an antipsychotic drug, is an example of an API which can hydrate into three different crystalline dihydrates (B, C and E) and a higher hydrate form [10]. This ability to form multiple hydrates makes it difficult to control the anhydrate to hydrate transformation during the process of wet extrusion. To the best of our knowledge, studies on the monitoring of the polymorphic transitions of olanzapine during the entire extrusion-spheronisation process have not been reported previously. Furthermore, there is a lack of information about how olanzapine behaves in moist environments and how excipients may control the transformations that are likely to take place during the pharmaceutical processes involving moisture.

This study aims (1) to access the time needed for olanzapine to undergo hydrate conversion when olanzapine comes in contact with moisture (which occurs during the second step of wet extrusion process); (2) to study whether excipients (e.g., PEG, HPC, PVP or MCC) can influence the hydrate conversions of olanzapine both during and after the wet massing step; (3) to characterize different hydrate crystal forms of olanzapine that may be generated during processing and (4) to find a formulation which allows the maintenance of the original crystal form of the API throughout the entire extrusion-spheronisation process. In particular, the understanding of the mechanisms underpinning the effect of polymeric excipients on the polymorphic conversion of olanzapine in aqueous environments was analyzed.

## 2. Results

**Figure 1:**
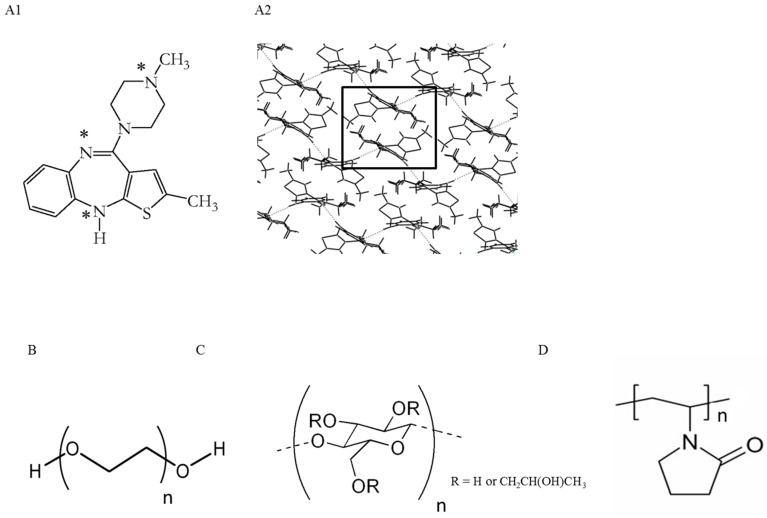
Olanzapine (OLZ) molecular structure (**A1**) and molecular packing (**A2**) of anhydrous OLZ FI and structures of PEGs (**B**); HPC (**C**) and PVPs (**D**). (*) Hydrogen bond acceptor or donor groups.

The different polymers used in this work may be classified into three categories according to their molecular structure. In group I polyethyleneglycol with different molecular weights (PEG 6000 and 40,000) were considered; this polymer has both hydrogen bond donor and acceptor groups at the two ends of the polymer chain (Figure 1). Group II is constituted by a cellulose derivative polymer, hydroxypropylcellulose (HPC) with both hydrogen bond donor and acceptor groups in their ring structure (Figure 1). Group III includes polyvinylpyrrolidone (PVP) which present only one acceptor hydrogen bonding group in their ring structure. Furthermore, these polymeric materials also present different surface free energies which affect their spreading over OLZ particles.

### 2.1. Polymer Screening Method to Identify Polymers that can Stabilize OLZ FI during Wet Massing

**Figure 2:**
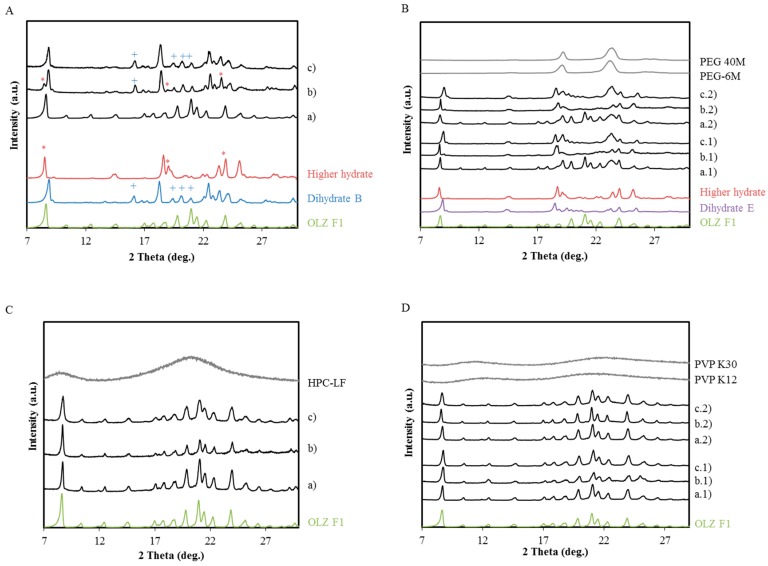
Diffractograms of six formulations before wetting, after wetting and after drying. The wet samples were analyzed after storage in a sealed container for 360 min. (**A**) OLZ before (a) and after wetting (b) and drying (c); (+) represents the characteristic peaks of the OLZ dihydrate B and (*) represents the characteristic peaks of the OLZ higher hydrate; (**B**) Formulations B1 and B2 physical mixture (a.1 and a.2), after wetting (b.1 and b.2) and after drying (c.1 and c.2); (**C**) Formulation C physical mixture (a), after wetting (b) and after drying (c); (**D**) Formulation D1 and D2 physical mixture (a.1 and a.2) after wetting (b.1 and b.2) and after drying (c.1 and c.2).

The X-ray powder diffraction (XRPD) patterns for OLZ, OLZ:Polymer physical mixtures and the respective wet/dried masses are represented in Figure 2. In order to characterize the changes in the crystal structure of OLZ in each formulation, the XRPD patterns were compared to the XRPD patterns of different crystal structures of OLZ, such as, anhydrous form I, dihydrate B, dihydrate E and higher hydrate.

Figure 2A shows the XRPD pattern of OLZ and recovered OLZ after 360 min of being in contact with water. The XRPD diffractograms show that OLZ was able to hydrate into the dihydrate-B form. However, the peaks at 8.58°, 19.20°, and 24.14° 2θ, which are characteristic of the higher hydrate form, suggest a concomitant crystallization of both hydrates, although in different proportions. The subsequent resting of the wet mass at 25 °C/55% RH for 24 h was followed by the elimination of the higher hydrate form. The higher hydrate is a metastable crystal form that contains 2–2.5 mol of water per mol of OLZ and has only been observed in wet cakes of OLZ [10], which explains its elimination after removal of the adsorbed water.

OLZ which was physically mixed with PEG 6000/PEG 40,000 was shown to hydrate selectively into the higher hydrate form (Figure 2B). The product from the dehydration of the higher hydrate (2.5 mol water per mol of OLZ) was the dihydrate E. The XRPD study of the wet material enabled the observation of the intermediate higher hydrate form which is generated and is only stable in a wet environment. Similar diffraction patterns of OLZ before and after wetting suggest a complete inhibition of hydrate transformations of OLZ during the time of the experiment. However, a slight widening of the OLZ peaks was observed after drying the OLZ: HPC wet mass (Figure 2C). Similarly, OLZ: PVP mixtures also failed to show transformations of OLZ. No peaks due to excipients were observed after the manufacturing of physical mixtures with PVP k12, PVP k30 and HPC-LF reflecting the amorphous nature of these polymers.

**Figure 3:**
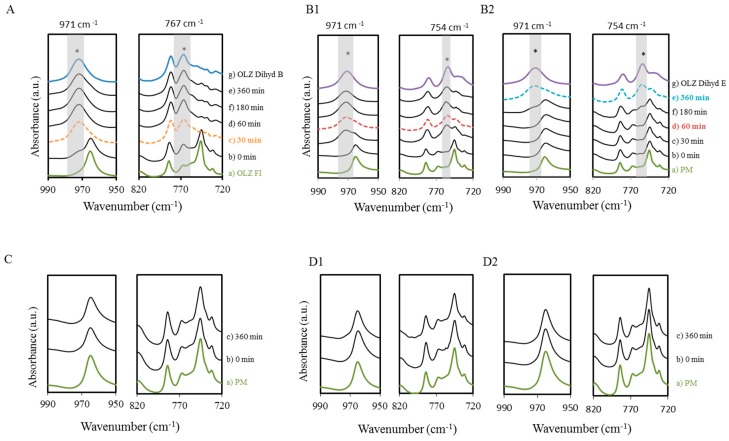
Spectra (FTIR) regions (950–990 cm^−1^ and 720–820 cm^−1^) of OLZ wet masses (1.5 OLZ:1 polymer) after storage in a sealed container. Formulations A (**A**); B_1_ (**B1**) and B_2_ (**B2**) show complete hydration after 30, 60 and 360 min (dashed lines); Formulations C (**C**); D_1_ (**D1**) and D_2_ (**D2**) show no hydration during the time of storage (360 min). (*) Represents the characteristic peaks of the OLZ dihydrate E.

FT-Infrared spectroscopy was also used to monitor the anhydrate to hydrate conversion of OLZ since the addition of water (wet massing, *t* = 0) until the end of the study (360 min) and the respective spectra are shown in Figure 3. The changes in the region 950–990 cm^−1^ confirmed the hydrate conversion independently of the type of hydrate formed, due to a shift on the bands at 965 cm^−1^. This shift was related to the deformation of the piperazinyl group coupled to the azepine and thiophene moieties (965 cm^−1^) in OLZ molecules [11]. Therefore, Figure 3 shows that hydrate formation of OLZ started at an early stage when polymers were absent from the formulation. This was visible by the shift of the bands at 965 cm^−1^ to 971 cm^−1^ for the samples taken immediately after the completion of the wet massing. For formulation B1 (OLZ:PEG 6000), the shift started after 30 min, showing a higher induction period for hydration of OLZ in the presence of this polymer. By increasing the polymer weight (B2), the induction time increased (Figure 3B2). In both PEGs’ wet masses, anhydrous OLZ was not visible after 360 min (Figure 3B1,B2). These results have shown that by using PEG as a binder it was possible to prevent hydration, provided the time allowed for OLZ to contact the aqueous solution was less than 30 min for PEG 6000 or less than 180 min for PEG 40,000. When PVPs and HPC replaced PEGs in the formulation, no signs of hydrate formation were observed during the period of the test (Figure 3C,D1,D2, 360 min).

The region 720–780 cm^−1^ was selected to distinguish the different hydrate conversion occurrences in OLZ wet masses containing no polymer (A) or the PEGs (B1 and B2) as excipients. The out of plane deformation of the CH bond of the benzene group at 745 cm^−1^ was no longer present in both wet masses after 360 min. Wet mass A revealed a new absorption maximum at 767 cm^−1^ specific for the dihydrate B whereas wet masses B1 and B2 presented a new band at 754 cm^−1^, characteristic of the dihydrate E.

**Figure 4:**
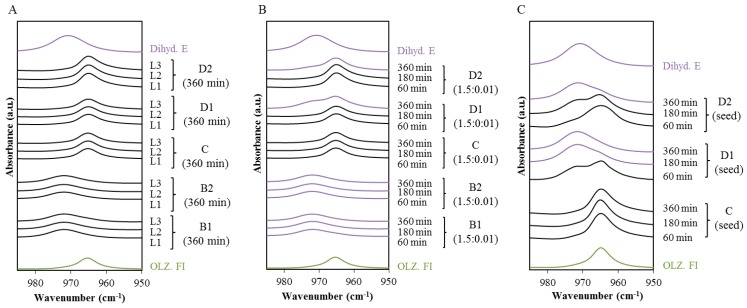
Spectra (FTIR) in the region (990–950 cm^−1^) for the different OLZ:polymer formulations (B1, B2, C, D1, and D2). (**A**) Formulations were wet massed with 3 levels of water L1 (50 g), L2 (75 g), L3 (100 g). The spectra were collected after 360 min mass preparation; (**B**) The polymer content had a two order of magnitude reduction (1.50 OLZ:0.01 Polymer); (**C**) Wet masses C, D1 and D2 with seeds (5%). The spectra were collected at 60, 180 and 360 min after the wet mass preparation.

Figure 4A displays the spectra within the range of 950–990 cm^−1^ of the wet masses (L1, L2 and L3, respectively 50, 75 and 100 g of water) collected 360 min after their preparation. Both PVP and HPC were shown to prevent hydrate formation regardless of the amount of water used. In Figure 4B, the fraction of the polymer was reduced by two orders of magnitude, in order to differentiate the ability of PVP and HPC to prevent hydrate conversion. The ratio of OLZ:Polymer was then fixed at 1.5:0.01. Figure 4B displays the spectra of the wet masses collected at three different times (*t* = 60, 180, 360 min) after their preparation. The reduction on the polymers fraction in formulations D was reflected by a reduction of the induction time for the hydrate conversion (especially for the formulation containing PVP k12), which started to hydrate after 360 min in a sealed container. Conversely, no hydrate transformation was observed in formulation C containing a low percentage of HPC in the wet mass.

Seeding experiments were carried out to determine if the HPC and PVP could still prevent the transformation in the presence of nuclei, and to better understand the inhibitory mechanism of these polymers. Five percent of the anhydrous API was thus replaced by OLZ dihydrate E before the wetting step. Shifts in the spectra of samples based on formulations D1 and D2 were observed and assumed to be related to hydration of anhydrous OLZ, whereas samples of formulation C did not produce shifts in the characteristic bands (Figure 4C).

**Figure 5:**
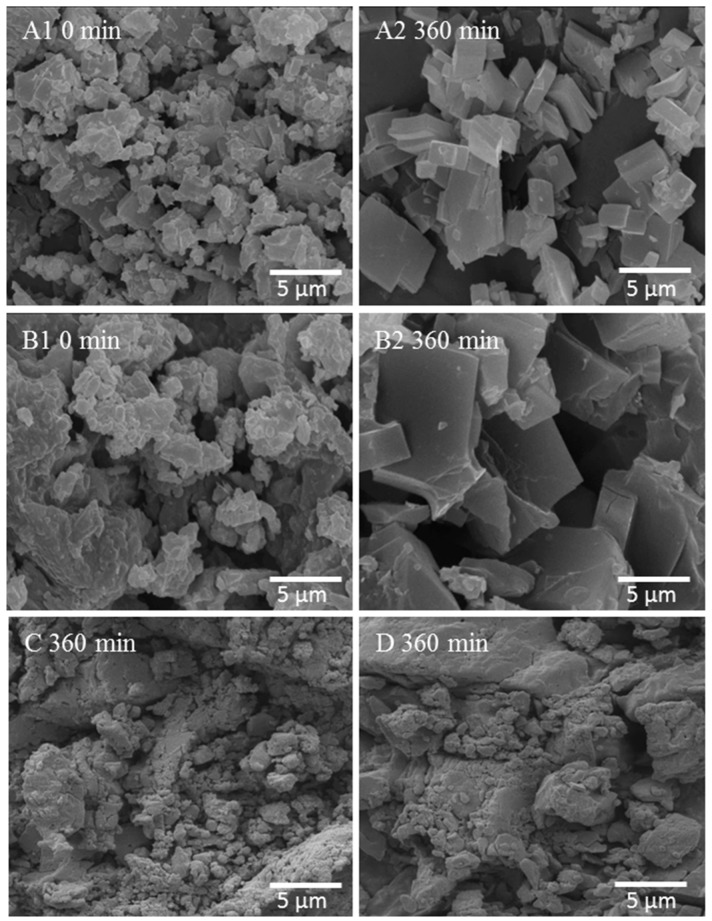
Micrographs of wet masses for formulations A, B_1_, C and D_1_. Formulation A (*t* = 0 min (**A1**) and *t* = 360 min (**A2**); Formulation B_1_ (*t* = 0 min (**B1**) and *t* = 360 min (**B2**)); Formulation C (*t* = 360 min (**C**)); Formulation D_1_ (*t* = 360 min (**D**)).

Figure 5 A1 shows that immediately after wet massing (*t* = 0), OLZ particles were irregularly shaped and agglomerated. OLZ particles, alone (A) or in the presence of PEG (B1), after 360 min in direct contact with water, had their morphology changed to plate-like shaped crystals (Figure 5 A2) characteristic of the hydrate and the size of the latter larger than OLZ particles alone (Figure 5 B2). Conversely, formulations C and D1 showed no transformation of OLZ particles over the same period (Figure 5 C,D).

**Figure 6:**
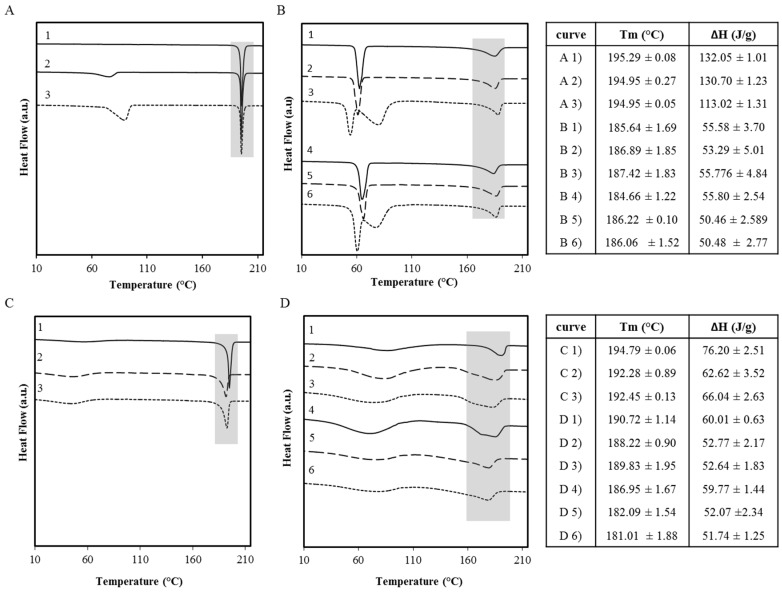
Thermograms of formulations A (**A**); B_1_ (**B**, curves 1–3; B_2_ (**B**, 4–6); C (**C**); D_1_ (**D**, 1–3) and D_2_ (**D**, 4–6). Curve 1 shows the physical mixture of raw materials, curves 2 and 5 show wet masses at *t* = 0 min, and curves 3 and 6 show the wet masses at *t* = 360 min. Tables present the melting point of OLZ and the respective enthalpy of fusion (Tmax and ΔH) for each formulation (grey shadow in figures).

The thermal behavior of anhydrous OLZ and physical mixtures of formulations B1, B2, C, D1 and D2 are shown in Figure 6 (line 1, A to D) in which thermograms 2 and 5 correspond to the samples recovered immediately after the wet massing (*t* = 0 min) and the curves 3 and 6 correspond to the samples which were kept in a sealed container (*t* = 360 min).

Figure 6A, curve 1 (OLZ raw-material) shows only one endotherm event at 195.29 °C due to the melting of OLZ. Figure 6A, curves 2 and 3 reveal a new endotherm at lower temperatures corresponding to the release of water in the molecular structure, as a result of the presence of OLZ hydrate in the samples. The small fraction of the hydrate content in OLZ samples which were produced immediately after the wet massing (a mixture of anhydrous and hydrous content is present, Figure 6) resulted in a water loss endotherm with a low enthalpy.

Figure 6B, curves 1 and 4 show that the physical mixtures (PM) obtained from formulations B_1_ and B_2_ present two endothermic peaks due to the melting of both PEG and OLZ. The depression on OLZ melting temperature and enthalpy results from the solubilization of the drug within the molten PEG during heating. Figure 6B, curves 2 and 5 show no traces of water evaporation allowing the assumption that no hydrate was present in the sample. However, curves 3 and 6 (Figure 6B) reveal an endothermic peak due to dehydration of the dihydrate E. Furthermore, a reduction in the melting temperature of the PEG polymers was also observed in the same thermograms, suggesting conformational changes of the polymer chains with an impact on their melting temperature. This is in line with the ability of the PEG chains to adapt their conformation when in contact with water to establish extensive hydrogen bonds [12]. In a different experiment, OLZ was heated up to 120 °C with dehydration, cooled and heated again (Figure 6B, curves 3 and 6). These samples revealed an increase of the PEG melting temperature in line with its initial value (data not shown). This finding suggests reversibility of the conformational changes of the polymer chains occurred after removal of water in the sample.

There was no apparent conversion to the OLZ dihydrate for the samples which were formulated in the presence of both PVP and HPC, however, some adsorbed water created a broad endotherm (Figure 6C,D). In thermograms C (curves C2 and C3) and D (curves D2, D3, D5 and D6), the OLZ endotherms due to melting became broader, with a lower enthalpy and temperature than those observed in the physical mixture (curve 1D). This shift towards lower temperatures indicates a higher miscibility of drug in the polymer after the wet massing process.

Table 1 shows the surface energy and the polarity for each polymer in the formulation. The results show the ranking for the measured hydrophobicity as HPC > PVP > PEG. Generally, for the binder (polymer) to spread over the substrate (API) in a wet massing process, the work of cohesion of the binder must be lower than that of the substrate, allowing the spreading of the first on the latter. This only occurred for HPC. These results were in line with the results obtained for the contact angles (Table 2) which have shown the wettability of the powders, as measured by the contact angle (°) of the substances in water. While the contact angle of the particles in formulations B1, B2, D1 and D2 after wet massing showed a contribution of the contact angles of both polymers and OLZ, the contact angle of particles in formulation C1 (OLZ with HPC) seemed to account only for the contribution of HPC (Table 2). Furthermore, a slightly lower contact angle was observed for these particles than for the isolated HPC suggesting that this polymer was not only completely covering the crystalline particles of OLZ, but also a decreasing number of hydrophobic groups were exposed on the surface of the particles, as a result of polymer chain conformation.

**Table 1:** Surface energy of olanzapine and polymers.

	γ^d^ ^1^ (mN/m)	γ^p^ ^1^ (mN/m)	γ ^1^ (mN/m)	Polarity (%)	W_c_^2^ (mJ/m^2^)	W_a_^2^ (mJ/m^2^)	W_a_−W_c_^3^ (mJ/m^2^)
OLZ	41.31	1.19	42.50	3	85.00		
PEG 6000	35.71	14.62	50.33	29	100.66	81.01	−19.65
PEG 40,000	34.78	12.93	47.71	27	95.42	79.89	−15.53
PVP k12	43.22	7.48	50.71	15	101.42	88.59	−12.83
PVP k30	40.46	6.73	47.19	14	94.38	85.81	−8.57
HPC LF	31.90	4.80	36.70	13	73.40	75.81	2.41

^1^ Surface free energy (γ) and its dispersive (γ^d^) and polar (γ^p^) components; ^2^ W_c_ and W_a_ are, respectively, the work cohesion and the work of adhesion (to OLZ); ^3^ W_a_–W_c_ reflect the spreading coefficient (λ).

**Table 2:** Contact angles of the raw materials and different formulations ^1^.

Raw-Material	Contact Angle (θ/°)	Formulations after Massing	Contact angle (θ/°)
OLZ	104.65 ± 0.64	A	104.87 ± 0.74
PEG 6000	63.60 ± 2.05	B1	81.24 ± 2.98
PEG 40,000	64.30 ± 1.95	B2	79.52 ± 1.07
HPC-LF	91.84 ± 0.47	C	87.41 ± 1.72
PVP k12	56.46 ± 1.36	D1	79.73 ± 1.54
PVP K30	75.77 ± 2.17	D2	89.14 ± 3.40

^1^ Contact angle measured in water (*n* = 6).

### 2.2. Characterization of OLZ Wet Masses and Pellets Containing Microcrystalline Cellulose

**Figure 7:**
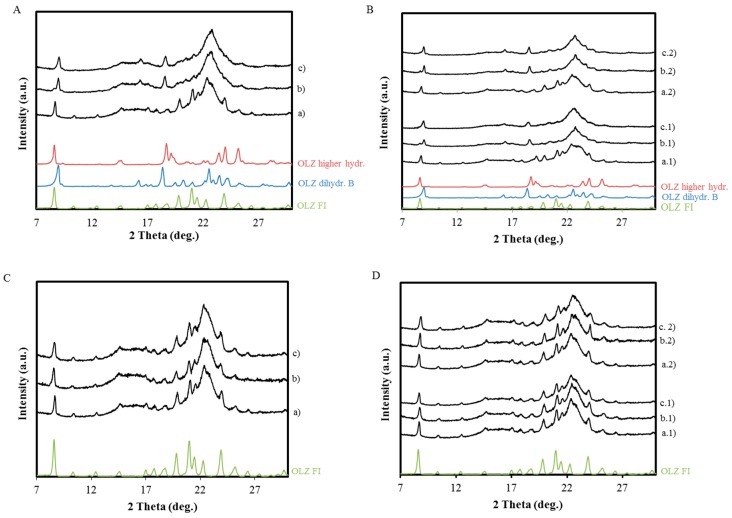
Diffractograms of formulations as physical mixtures (a), as wet masses (*t* = 360 min), (b) and dried pellets (c). (**A**) Formulation A_M_; (**B**) Formulation B1_M_ (a.1–c.1) and B2 _M_ (a.2–c.2); (**C**) Formulation C_M_; (**D**) Formulation D1_M_ (a.1–c.1) and D2_M_ (a.2–c.2).

For the formulations containing MCC, the ratio OLZ:polymer was maintained, as well as the amount of liquid used for the wet massing. The examination of the diffractograms acquired off-line after the wet massing (*t* = 360 min) (Figure 7) revealed that the contact with the liquid binder triggered the hydrate formation in formulations A_M_, B1_M_ and B2_M_ (Figure 7A,B). In all these formulations, both OLZ dihydrate B and OLZ higher hydrate were observed in the wet mass. For formulations B1_M_ and B2_M_, the conversion of the anhydrous OLZ into two hydrates, instead of only one hydrate (B1 and B2), suggests that PEG was not interacting with the drug at the same extent as without the MCC. Drying the pellets (Figure 7Ac,Bc1,c2) at 45 °C for 60 min was not sufficient to remove the dihydrate B, which was visible in both OLZ formulations (Figure 7Ac,Bc1,c2).

**Figure 8:**
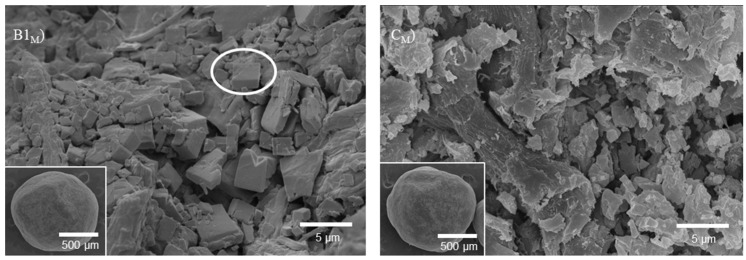
Micrographs (SEM) of a cross section and pellets (inserts) from formulations B_1M_ (**left**) and C_M_ (**right**). The circle in the left micrographs shows a crystal of OLZ dihydrate.

Figure 8 shows a cross section of the pellets B1_M_ and C_M_. The appearance of plate-like crystals of OLZ in the inner part of the pellet has shown that the changes in shape of OLZ particles due to hydration were also occurring in the formulations containing MCC.

**Figure 9:**
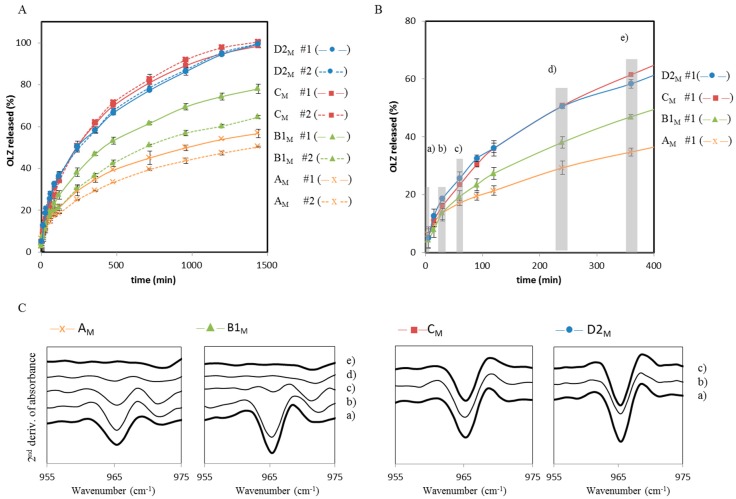
Dissolution profiles of OLZ from pellets (top) and Spectra (FTIR) analysis made from different formulations (A_M_, B1_M_, C_M_ and D2_M_). (**A**) Dissolution profiles of OLZ from pellets #1 (*t* = 0 min) and #2 (*t* = 360 min); (**B**) Formulations and the time periods (a–e) in which pellets were collected for FTIR characterization; (**C**) Second derivative of FTIR absorbance spectra from collected samples (times a–e for formulations A_M_ and B1_M_ and times c–d for formulations C_M_ and D2_M_).

Figure 9a shows the dissolution curves of OLZ released from pellets prepared immediately before the test. For each formulation, dissolution profiles were obtained from A_M_, B1_M_ (PEG 6000), C_M_ (HPC) and D2_M_ (PVP k30) pellets made from non-stored (#1, *i.e.*, *t* = 0) and stored (#2, *i.e.*, *t* = 360 min) wet masses. A significant change on OLZ dissolution rate (*p* < 0.05) was observed between #1 and #2 pellets for A_M_ and B1_M_ formulations. On the contrary, no significant differences in the OLZ dissolution rate from pellets #1 and #2 were observed from formulations C_M_ (HPC) and D2_M_ (PVP), although a slight increase in OLZ dissolution rate was verified for pellets #2.

During dissolution, it was possible to characterize the OLZ solid-state form remaining in the pellets. The second derivative from FTIR absorbance spectra of the pellets taken from the dissolution media has shown that PVP and HPC can also have a suppressing effect on the OLZ hydrate conversion in the dissolution media, by opposition to pellets A_M_ (no polymer) and B1_M_ (PEG) in which OLZ suffered hydration. This observation strongly suggests that HPC and PVP, which had shown a strong protection of OLZ over the production of wet masses, also stabilize the anhydrous OLZ during their release from pellets into the dissolution media.

Table 3 summarizes the results of OLZ release modelling for the pellets under investigation. The goodness of fit for the various models were ranked in the order: Korsmeyer-Peppas > Higuchi > First-order for the formulations A_M_, B1_M_ and C_M_. For formulation D2_M_, the goodness of fit for the first-order model was better than for the Higuchi model. The fact that drug release from pellets follows Korsmeyer-Peppas model and the values of the exponent *n* are lower than 0.5 (0.383–0.441) indicates that the OLZ, independently of the polymorphic form present in the pellets, follows a diffusion controlled release.

**Table 3:** Fitting of dissolution models to OLZ release data for pellets.

Formulation		First-Order Model			Higuchi Model			Korsmeyer-Peppas Model			
		Adj R^2^	MSE Root	K_1_	Adj R^2^	MSE Root	k_H_	Adj R^2^	MSE Root	k_KP_	*n*
A_M_	#1	0.669	9.946	0.001	0.952	3.797	1.648	0.997	0.958	3.585	0.383
	#2	0.706	8.624	0.001	0.97	2.728	1.441	0.995	1.127	2.676	0.407
B1_M_	#1	0.82	10.15	0.002	0.962	4.674	2.218	0.994	1.794	4.442	0.395
	#2	0.839	8.466	0.001	0.986	2.464	1.821	0.995	1.554	2.703	0.441
C_M_	#1	0.951	6.910	0.002	0.986	3.81	2.696	0.995	2.226	4.450	0.431
	#2	0.926	8.408	0.003	0.973	5.044	2.755	0.995	2.353	4.966	0.411
D_M_	#1	0.972	5.697	0.003	0.958	6.991	2.995	0.980	4.801	5.510	0.408
	#2	0.978	5.115	0.003	0.956	7.321	3.069	0.981	4.658	5.494	0.410

#1 and #2 represent pellets manufactured at *t* = 0 min and *t* = 360 min of wet masses, respectively.

## 3. Discussion

The different crystalline forms of OLZ are built through the assemblage of (OLZ) centrosymmetric racemic pairs and then connected to each other through hydrogen bonds, either from atoms like in the OLZ structure (e.g., anhydrous form) or by water molecules (e.g., hydrate forms). In form I, the hydrogen bond interactions between the amine and imine (NH-N) are responsible for the connection of two different OLZ pairs [10]. When the hydrate formation occurs, the NH–N interactions are disrupted and replaced by water bridging molecules between the dimmers. The presence of water balances the hydrogen bond donor/acceptor ratio of OLZ, thus allowing two acceptors to participate in hydrogen-bonding, which was not possible in the anhydrates. Therefore, in OLZ hydrates, a single hydrogen-bond donor (NH) and two good acceptors (imine and piperidine) may participate in hydrogen bonding with water.

In manufacturing processes in which water is added (e.g., wet extrusion), OLZ is exposed to conditions in which the hydrate forms are expected to be the most stable due to high water activity [13]. Consequently, starting from the addition of water until the final drying, the API may undergo an anhydrate to hydrate transformation which may or, may not, be completed by the end of the process. The API transformations in an aqueous environment are expected to occur via a solvent-mediated transformation (SMT) [13] which encompasses a three-stage process. Firstly, the metastable phase in this work, the anhydrous OLZ, starts to dissolve. Since the dissolution of the metastable phase is a prerequisite for the transformation to take place, the solubility of the metastable form will initially provide the thermodynamic driving force for the phase transformation. Dissolution under non-sink conditions will lead to a supersaturation with respect to the stable hydrate phase and provide a thermodynamic driving force for nucleation of the stable phase (hydrate). The next stage involves nucleation of the stable hydrate once supersaturation has been reached. During wet massing of powders, which can be regarded as a suspension, there is an abundance of particles in the form of the anhydrate phase, which might aid heterogeneous nucleation [14]. By opposition to homogeneous nucleation, heterogeneous nucleation lowers the thermodynamic free energy barrier. Thus, heterogeneous nucleation can occur under lower driving forces [15]. Finally, once nuclei of the stable form are present in the solution, growth of that form continues until all the metastable phase has dissolved. Therefore, the growth of the stable phase (hydrate) is maintained by the supersaturation created by the dissolution of the metastable phase [14,16]. The kinetics of solvent-mediated transformations is therefore governed by dissolution of the unstable phase and nucleation and growth of the stable phase. It follows that, if nucleation of the hydrate phase could be inhibited by polymers, then crystal growth could not occur, *i.e.*, inhibition of crystal growth only became important if seeding crystals were present. When observing the XRPD data obtained from the masses without the presence of excipients, one could observe that a concomitant crystallization of both dihydrate B and higher hydrate took place on the OLZ wet mass A. This phenomenon may be associated with the nucleation rates, favoring the concomitant crystallization of both hydrates [15]. Also, it was noted that the hydration of all of the material was not finished within the time scale of the wet massing step. The kinetics of this process was controlled by the dissolution rate of the unstable form, the rate of crystallization of the more stable form and their relative solubilities. It means that in wet massing, the extent of hydrate formation is proportional to the solubility of the anhydrate and the volume of the wetting liquid used [17]. Furthermore, since wetting of powders is a factor governing hydrate formation kinetics [18], it is clear that the APIs with poor wettability will require an excessive amount of moisture and/or prolonged contact with wet massing liquid for the hydrate to form. Thus, the short wet massing period together with the low wettability of OLZ (θ ≈ 104°) led to the incomplete hydrate conversion of OLZ during wet massing. Furthermore, its low solubility (0.043 mg/mL [19], *i.e.*, a small quantity of OLZ can be dissolved in the liquid at any time) resulting in a slower hydrate rate conversion, which contrasts with other drugs such as theophylline (solubility in water of 12 mg/mL) [20] or sulfaguanidine (solubility in water of 1.38 mg/mL) that was shown to hydrate in less than 5 min during wet granulation processes [13].

It has been reported that the inhibition of polymorphic transitions by the polymers is related to their hydrophobicity, viscosity and steric hindrance. Polymers with higher hydrophobicity (lower polarity) are less likely to be present in the aqueous phase rather than being adsorbed on the API particles’ surface, inhibiting therefore the API interaction with the water molecules [13]. The addition of PEG 6000 and 40,000 to the formulations, respectively B1 and B2, has retarded the hydrate conversion without concomitant crystallization into two different hydrates. In these wet masses, the OLZ higher hydrate crystallized as a single metastable form that was shown to dehydrate easily into the dihydrate E form when the samples were dried. The addition of PEG 40,000 (higher viscosity) was shown to increase the lag time for hydration in comparison with PEG 6000. The rate by which the new crystals are formed in supersaturated solutions is related to the diffusion rate of the drug molecules [14]. The addition of PEG might increase the viscosity of the media affecting the drug’s diffusivity, which in turn affects the dissolution, nucleation and crystal growth rates [21]. The change of the nucleation kinetics caused by PEG favored the appearance of a single form—higher hydrate—which is never obtained as an isolated form in water environments without any additive. On the other hand, a complete inhibition of OLZ hydration was achieved by addition of both HPC and PVP. According to the literature, there are two major factors that lead to an efficient hindrance of the hydrate formation by the polymeric excipients: the capacity to form hydrogen bonds with the drug and a sufficient hydrophobicity. Both these features prevent the drug from binding with water and force it to bind with the polymer instead [9,13,22], which are important factors affecting the nucleation and growth rates of crystals.

For nucleation to occur, drug-polymer hydrogen bonds have to be broken so that drug molecules may diffuse and form a nucleus. In the wet masses with HPC (C) and PVP (D), OLZ molecules may interact with the polymer molecules to form OLZ-polymer hydrogen bonds in the aqueous environment. These interactions may then inhibit or retard the formation of nuclei due to collisions. The strength of the drug-polymer association determines the time required for the nucleation to take place [13].

When the content of HPC, PVP k30 and PVP k12 was reduced by two orders of magnitude in formulations C, D1 and D2, respectively, only HPC was shown to continue to inhibit hydrate conversion. This can easily be explained by the hydrogen’s bond ability of the involved polymer and its spreading over OLZ particles. While HPC has both hydrogen bond donor and acceptor groups in its ring structure, PVP has just one hydrogen bond acceptor group per monomer unit. In a different experiment, where hydrate seeds were added to the wet masses, transformation times decreased for PVPs. When hydrate seeds were added to the HPC formulation, the hydrate transformation continued to be inhibited during the 6 h of the test, indicating that HPC can effectively inhibit the crystal growth. The stronger inhibition of hydration by HPC may be justified by the higher H-bond ability and higher network association of this polymer compared to PVP, which could have contributed to a stronger bond with the crystal surfaces of the hydrate nuclei/initial crystals and promote steric hindrance to the incorporation of other drug molecules into the crystal lattice, thus preventing the crystal growth. Although PVP was not successful in preventing transformations, polymer molecular weight had an impact (formulations D1 and D2). In both cases, the nucleation step was no longer a limiting factor but nuclei grew at different rates: faster for PVP k12 than for PVP k30. This was also observed by other authors [9] for which the inhibition effect of polymers on carbamazepine was also related to their hydrophobicity, since polymers with a higher hydrophobicity had a stronger interaction with the carbamazepine crystal surface. Data has shown that HPC had a lower polarity (higher hydrophobicity) and a lower cohesion ability than its ability to adhere to the OLZ molecules. This showed the ability of this polymer to spread over the OLZ crystal surfaces.

Moreover, the OLZ powders, which were subjected to wet massing with HPC, showed that this polymer can reduce the contact angle of OLZ to the same values than the polymer alone, revealing that the polymer could effectively spread on and bind to the crystal surface of OLZ particles. The other more hydrophilic polymers were not so successful in spreading on OLZ surfaces. These results indicate affinity of this polymer to OLZ surfaces, which when absorbed at relevant growth sites, may have led to a blockage of the growth of the nuclei.

The addition of cellulose to the wet masses as an excipient did not have an effect on the inhibition of hydrate conversion, and the respective formulations A_M_, B1_M_ and B2_M_, OLZ were shown to hydrate in the same manner as had occurred in formulations without HPC or PVP. However, while in formulation B1 and B2 only one hydrate was obtained (higher hydrate), in formulations with cellulose (B1_M_ and B2_M_), two hydrates were visible in the wet mass (dihydrate B and higher hydrate). This loss of selectively of hydrate conversion indicates a reduction of the close contact between the polymer (PEG) and the drug.

A better understanding of the dissolution process can be accomplished by analyzing not only the drug in the liquid dissolution medium but also in the solid state of the dissolving sample, which provides a more complete understanding of the dissolution behavior.

The fast and similar dissolution rate of OLZ present in all the different pellets formulations at the very beginning can be partly explained by the dissolution of OLZ anhydrate before the hydrates began to crystallize. Moreover, the dissolution of OLZ particles on the surface of the pellet can promote the burst effect during the first few minutes.

The mechanism by which OLZ hydrates in the dissolution vessel is expected to be the same as the one that occurs when OLZ is subjected to the wet massing process, since in this situation the drug is also exposed to a solvent. Aaltonen *et al.* [23] reported hydration by solvent-mediated phase transformation of theophylline and nitrofurantoin during dissolution in purified water. The FTIR absorbance spectra of OLZ present in the pellets after being for different periods in the dissolution medium (not dissolved OLZ fraction) showed that both PVP and HPC had a suppressing effect on the OLZ hydrate formation during the dissolution test, which coincides with the best performance of OLZ.

During the dissolution process of the pellets, several processes competing with each other may take place simultaneously, such as: dissolution of OLZ, recrystallization of anhydrous OLZ to OLZ hydrated, swelling of the polymer and dissolution of the polymer. The polymers that were shown to strongly interact with the drug during the wet massing of OLZ may also stabilize the anhydrous OLZ during the dissolution by inhibiting aggregation due to a stronger interaction between the drug and polymer in the dissolution media. Therefore, the interaction with the polymers prevents the formation of the hydrate crystals, thus rendering the drug dispersed in the matrix of the polymer easier to dissolve.

## 4. Experimental Section

### 4.1. Materials

Olanzapine anhydrous form I [OLZ FI, molecular weight (Mw) = 312.43 g/mol, density (d) = 1.30 gcm^−3^] was purchased from Pharmorgana, India. The OLZ molecule and representation of its three-dimensional structure of anhydrous OLZ form I is represented in Figure 1.

The polymers considered in the study were polyethyleneglycol with different molecular weights (PEG, Mw = 6000 and 40,000, Sigma-Aldrich, Munich, Germany), hydroxypropylcellulose (HPC LF Pharm grade, Mw = 95,000, Klucel™, Ashland, Düsseldorf, Germany) and polyvinylpyrrolidone (PVP, Kollidon 12, 2600 < Mw < 5500 and Kollidon 30, 44,000 < Mw < 54,000, BASF Chemicals, Ludwigshafen, Germany). Other chemical were supplied by Sigma-Aldrich, Munich, Germany.

The following OLZ forms were produced in order to identify the transformations that OLZ FI may undergo during processing:

#### 4.1.1. OLZ Dihydrate B

(CSD ref. code: AQOMAU01, [10]): OLZ Form I (2.5 g) was suspended in ethyl acetate (25 mL) and toluene (2 mL) under stirring before heating (80 °C) for complete dissolution. Then, the solution was cooled down to 60 °C prior to the addition of water (30 mL), and further cooled down to room temperature, producing a crystalline slurry. The crystals of the dihydrate B were collected by filtration under vacuum;

#### 4.1.2. OLZ Dihydrate E

(CSD ref. code: AQOMAU02, [10]): OLZ Form I (3 g) was suspended in ethyl acetate (60 mL) and toluene (3.6 mL). The suspension was stirred and heated to 80 °C for complete dissolution. The solution was then cooled to ambient temperature to produce a slurry. The suspension was filtered, washed with water and dried at room temperature.

#### 4.1.3. OLZ Higher Hydrate

(CSD ref. code: AQOMEY, [10]): OLZ (2 g) was suspended in CH_2_Cl_2_ (12 mL). The suspension was stirred and heated to reflux to dissolve the solids. Water (1.5 mL) was added as the solution was cooled to ambient temperature, at which time the crystal slurry formed. The crystal slurry was cooled to 0 °C, and a wet cake (2.7 g) was isolated by vacuum filtration and washed with CH_2_Cl_2_.

### 4.2. Methods

#### 4.2.1. Conversion of OLZ FI during Wet Massing in the Presence of Polymers

Four wet masses were prepared using anhydrous OLZ form I (OLZ FI, 15 g, “*formulation A*”) or physical mixtures of OLZ with each one of the different polymers commonly used in extrusion processes [24,25], belonging to the three groups described above, namely PEG-6000/ PEG 40,000 (group I, “*formulation B1 and B2*”, 10 g), HPC-LF (group II, “*formulation C*”, 10 g) and PVP k12/ PVP k30 (group III, *“formulation D1 and D2*”, 10 g) (Table 1). OLZ or physical mixtures of OLZ and polymer (1.5:1) were blended for 5 min in a planetary mixer (Kenwood, Hampshire, UK). Demineralized water (75 g, 5 times the OLZ mass) was added for 1 min to the blends. The wet masses were mixed thoroughly for 10 min and then placed into sealed glass vials filled to the top. Samples were taken at different time periods (*t* = 0, 30, 60, 180 and 360 min) after production. A fraction of each sample was analyzed immediately by XRPD and the remaining portion was dried in Petri dishes (24 h at 25 °C/55% RH, until constant weight) and analyzed by infrared spectroscopy (FTIR), thermal analysis (calorimetry, DSC) and microscopy (SEM). These formulations (B1, B2, C, D1 and D2) and processing conditions took into consideration other quantities of water, namely 50 and 100 g and, fractions of polymer, ranging from 1.5:1 to 1.5:0.01.

In a different set of experiments, 5% of anhydrous OLZ was replaced by OLZ dihydrate, to promote crystal growth within the anhydrous OLZ containing formulations.

#### 4.2.2. Manufacture of Pellets Containing Polymers

Taking into consideration the same fractions of both API and excipients, 75 g MCC was added to the blends described previously (Table 4), to turn the manufacture of pellets possible: OLZ:MCC (A_M_), OLZ:MCC:PEG-6000/40,000 (B1_M_ and B2_M_, respectively), OLZ:MCC:HPC (C_M_) or PVP k12/k30 (D1_M_ and D2_M,_ respectively) were dry-mixed in a planetary mixer (Kenwood, UK) for 5 min prior to the addition of 75 g (5 times the olanzapine mass) of demineralized water. Each wet mass (175 g) was further mixed for another 10 min. Half of the wet mass was extruded immediately (#1) whereas the other half was allowed to equilibrate for 360 min (#2) in a sealed polyethylene bag before extrusion. The storage of wet masses to equilibrate for several hours is commonly considered in daily practice to ensure a uniform distribution of water [26,27] and in the present case to promote hydration of OLZ. All measurements were performed off-line at defined time points after wet massing (*t* = 0, 30, 60, 180, and 360) by FTIR and XRPD.

Extrudates were manufactured from 2 wet masses (#1 and #2) in a ram extruder (Lurga, Portugal) adjusted to an universal testing machine (LR 50K, Lloyds Instruments, UK) fitted with a load cell to allow the collection of data for the applied force to the ram and its displacement (200 mm/min). The wet mass was forced to pass through a die (Length/Diameter = 6) and, after extrusion, the extrudates were immediately placed in a spheroniser (radial friction plate rotating at 500 rpm, Caleva, UK) for 5 min. The wet pellets were dried in a fluid bed drier (Aeromatic-Fielder AG, Bubendorf, Switzerland) at 40 °C for 30 min. Dried pellets were evaluated for powder diffraction (XRPD) infrared spectroscopy (FTIR) and OLZ dissolution studies into an aqueous solution.

**Table 4:** Formulations considered in the manufacture of pellets ^1^.

Formulation	Components (in Parts)		
	OLZ	Polymer	MCC
A_M_	1.5	-	8.5
B_1M_	1.5	1.0 (PEG 6000)	7.5
B_2M_	1.5	1.0 (PEG 40,000)	7.5
C_M_	1.5	1.0 (HPC-LF)	7.5
D_1M_	1.5	1.0 (PVP k12)	7.5
D_2M_	1.5	1.0 (PVP k30)	7.5

^1^ These formulations were compared to the respective ones without MCC (e.g., C (1.5 OLZ:1 HPC-LF) *vs.* C_M_ (1.5 OLZ:1HPC-LF:7.5 MCC)).

#### 4.2.3. Characterization of Blends and Pellets

##### X-ray Powder Diffraction (XRPD)

The structural characterization of OLZ formulations were carried out by X-ray powder diffraction (XRPD) on an Analytical X’Pert PRO apparatus set with a vertical PW 3050/60 goniometer (θ–2θ mode) equipped with X’Celerator detector with automatic data acquisition (X’Pert Data Collector (v2.0b) software), using a monochromatized CuKα radiation as incident beam, 40 kV–30 mA. Diffractograms were obtained by continuous scanning in a 2θ range of 7–35° with a step size of 0.017° and scan step times of 20 s.

X-ray diffraction characterization was performed to the different formulations (A, B1, B2, C, D1, D2) before and after the end of the study (360 min) in order to observe the hydrate conversions of OLZ in each sample.

##### Differential Scanning Calorimetry (DSC)

Thermograms of different OLZ forms were obtained with a Differential Scanning Calorimeter (TA instruments, Q200, New Castle, DE, USA) after calibration with indium (TA instruments; T_fus_ = 156.55 °C, Δ_fus_H = 28. 51 J/g). Dry N_2_ was used as the purge gas (Air Liquide, 50 mL/min). Dried samples (2–3 mg) were placed in pinhole crucibles and heated at 10 °C/min within 0–210 °C temperature range.

##### FT-Infrared Spectroscopy (FTIR)

Each sample was mixed with KBr in a 1:1 proportion and the mixture (200 mg) compacted into compacts (12 mm diameter). The infrared spectra were collected in the absorption mode (IR Affinity-1 Shimadzu spectrophotometer, Kyoto, Japan) based on 64 scans with a resolution of 2 cm^−1^.

##### Scanning Electron Microscopy (SEM)

OLZ wet masses and pellets were analyzed by scanning electron microscopy. The samples were mounted on aluminum stubs and coated with gold by ion sputtering (JEOL JFC-1200 Fine Coater, Tokyo, Japan) and observed under (JSM-5200 LV, Tokyo, Japan) scanning electron microscope.

##### Wettability and Surface Energy Measurements

In order to measure the wettability and the surface energy of the powders before and after being exposed to hydrating conditions, the contact angles in two liquids (bi-distilled water, Ström and diiodometane) of the samples were measured (Wilhelmy plate technique, Krüss Tensiometer K12, Hamburg, Germany). Samples of powders were made to adhere on a rectangle shape substrate (20 × 20 mm) uniformly coating its surface. All measurements were performed at a controlled temperature 25 ± 0.5 °C, by flowing water from a circulator (Krüss). The surface energy of the polymers and OLZ was measured according to the Wu equation [28].

##### Dissolution Studies

Dissolution studies were performed for OLZ (5 mg) from dried pellets (60 min/45 °C, size of 1–1.4 mm). Tests were performed in a dissolution apparatus (paddle method, 50 rpm, 900 mL of phosphate buffer at 6.8, 37 ± 0.5 °C, *n* = 6, AT7, SOTAX, Aesch, Switzerland) with collection of samples at time intervals (*t* = 5 to 1440 min). The residual water present in the pellets was taken into consideration (Sartorius Moisture Analyser, Goettingen, Germany). OLZ was quantified by UV spectrophotometry (λ = 254 nm, Hitachi U-2000, Tokyo, Japan) and the analysis of OLZ release and dissolution profiles was possible by comparison of dissolution models. Simultaneously, dissolution tests were performed with pellets A_M_, B1_M_, C_M_ and D2_M_ for off-line FT-Infrared evaluation of the anhydrate to hydrate transformations occurring in non-dissolved fraction of OLZ present in the pellets (*t =* 0 min, 30 min, 60 min, 180 min and 360 min). Each sample collected for FT-IR analysis was recovered from a different vessel.

## 5. Conclusions

In an attempt to clarify the mechanisms underlying the effect of polymeric excipients on the hydrate conversion of anhydrous OLZ in aqueous environments, the influence of various polymers on the hydration of OLZ in wet masses was investigated. The polymer screening methodology used to identify polymers that could stabilize OLZ during wet massing proved to be useful to identify which polymers may have the strongest effect of inhibition of OLZ. For the polymers selected in this study, it was found that both hydrogen bond ability and hydrophobicity (with impact on the spreading ability of the polymer) had an impact on the inhibition ability of OLZ hydration. In general all polymers promoted the inhibition of the transformation with different degrees of success, provided the contact time of OLZ anhydrous with water was low (e.g., less than 30 min). PEGs delayed the conversion of OLZ, but after 360 min of storage in a moist environment, conversion was observed. The HPC had the strongest effect on protecting the hydrate conversion of OLZ. The major reduction of the wettability of the OLZ powders in a blend with HPC revealed that this polymer can adsorb to OLZ’s surface inhibiting the water attack and, therefore, causing a hydrate formation. The presence of microcrystalline cellulose in formulations, needed for the pellets production, neither improves the protection against water nor did it eliminate the protector effect of both PVP and HPC. Conversion of the anhydrous form into hydrate was partially reversible and occurred during the wetting, drying and dissolution test in which the anhydrous form was converted into a hydrate. The combination of data retrieved from the different analytical techniques (FTIR spectroscopy, XRPD, calorimetry and microscopy) was successfully used to investigate the hydrate conversion of OLZ in various excipients allowing to visualize the different hydrate conversions that OLZ may undergo in the presence of excipients.

## Abbreviations

API	Active pharmaceutical ingredient
CSD	Cambridge structural database
DSC	dynamic scanning calorimetry
*Formulation A* (A)	OLZ + water
*Formulation B1/B2* (B1/B2)	OLZ + (PEG 6000/PEG 40,000) + water
*Formulation C* (C)	OLZ + HPC + water
*Formulation D1/D2* (D1/D2)	OLZ + (PVP k12/PVP k30) + water
*Formulation A_M_* (A)	OLZ + water + MCC
*Formulation B1_M_/B2_M_* (B1_M_/B2_M_)	OLZ + (PEG 6000/PEG 40,000) + water + MCC
*Formulation C_M_* (C_M_)	OLZ + HPC + water + MCC
*Formulation D1_M_/D2_M_* (D1_M_/D2_M_)	OLZ + (PVP k12/PVP k30) + water + MCC
FTIR	Fourier transformed infrared spectroscopy
HPC	Hydroxypropylcellulose
HPMC	Hydroxypropylmethylcellulose
MCC	Microcrystalline cellulose
OLZ	Olanzapine
PVP	Polyvinylpyrrolidone
SEM	Scanning electronic microscopy
SMT	Solvent-mediated polymorphic transformations
XRPD	X-ray powder diffraction

## References

[1] Phadnis N.V., Suryanarayanan R. (1997). Polymorphism in anhydrous theophylline—Implications on the dissolution rate of theophylline tablets. J. Pharm. Sci..

[2] Airaksinen S., Luukkonen P., Jorgensen A., Karjalainen M., Rantanen J., Yliruusi J. (2003). Effects of excipients on hydrate formation in wet masses containing theophylline. J. Pharm. Sci..

[3] Patel D.D., Anderson B.D. (2015). Adsorption of Polyvinylpyrrolidone and its Impact on Maintenance of Aqueous Supersaturation of Indomethacin via Crystal Growth Inhibition. J. Pharm. Sci..

[4] Gift A.D., Luner P.E., Luedeman L., Taylor L.S. (2008). Influence of polymeric excipients on crystal hydrate formation kinetics in aqueous slurries. J. Pharm. Sci..

[5] Sandler N., Rantanen J., Heinamaki J., Romer M., Marvola M., Yliruusi J. (2005). Pellet manufacturing by extrusion-spheronisation using process analytical technology. AAPS PharmSciTech.

[6] Jorgensen A., Rantanen J., Karjalainen M., Khriachtchev L., Rasanen E., Yliruusi J. (2002). Hydrate formation during wet granulation studied by spectroscopic methods and multivariate analysis. Pharm. Res..

[7] Dukić-Ott A., Thommes M., Remon J.P., Kleinebudde P., Vervaet C. (2009). Production of pellets via extrusion-spheronisation without the incorporation of microcrystalline cellulose: A critical review. Eur. J. Pharm. Biopharm..

[8] Katzhendler I., Azoury R., Friedman M. (1998). Crystalline properties of carbamazepine in sustained release hydrophilic matrix tablets based on hydroxypropyl methylcellulose. J. Control. Release.

[9] Tian F., Saville D.J., Gordon K.C., Strachan C.J., Zeitler J.A., Sandler N., Rades T. (2007). The influence of various excipients on the conversion kinetics of carbamazepine polymorphs in aqueous suspension. J. Pharm. Pharmacol..

[10] Reutzel-Edens S.M., Bush J.K., Magee P.A., Stephenson G.A., Byrn S.R. (2003). Anhydrates and Hydrates of Olanzapine: Crystallization, Solid-State Characterization, and Structural Relationships. Cryst. Growth Des..

[11] Ayala A.P., Siesler H.W., Boese R., Hoffmann G.G., Polla G.I., Vega D.R. (2006). Solid state characterization of olanzapine polymorphs using vibrational spectroscopy. Int. J. Pharm..

[12] Thijs H.M.L., Becer C.R., Guerrero-Sanchez C., Fournier D., Hoogenboom R., Schubert U.S. (2007). Water uptake of hydrophilic polymers determined by a thermal gravimetric analyzer with a controlled humidity chamber. J. Mater. Chem..

[13] Gift A.D., Luner P.E., Luedeman L., Taylor L.S. (2009). Manipulating hydrate formation during high shear wet granulation using polymeric excipients. J. Pharm. Sci..

[14] Wikström H., Rantanen J., Gift A.D., Taylor L.S. (2008). Toward an Understanding of the Factors Influencing Anhydrate-to-Hydrate Transformation Kinetics in Aqueous Environments. Cryst. Growth Des..

[15] Rodriguez-Hornedo N., Murphy D. (1999). Significance of controlling crystallization mechanisms and kinetics in pharmaceutical systems. J. Pharm. Sci..

[16] Mangin D., Puel F., Veesler S. (2009). Polymorphism in Processes of Crystallization in Solution: A Practical Review. Org. Process Res. Dev..

[17] Davis T.D., Peck G.E., Stowell J.G., Morris K.R., Byrn S.R. (2004). Modeling and monitoring of polymorphic transformations during the drying phase of wet granulation. Pharm. Res..

[18] Otsuka M., Ishii M., Matsuda Y. (2002). Effect of surface-modification on hydration kinetics of nitrofurantoin anhydrate. Colloids Surf. B.

[19] Thakuria R., Nangia A. (2011). Polymorphic form IV of olanzapine. Acta Crystallogr. Sect. C Cryst. Struct. Commun..

[20] Wikstrom H., Carroll W.J., Taylor L.S. (2008). Manipulating theophylline monohydrate formation during high-shear wet granulation through improved understanding of the role of pharmaceutical excipients. Pharm. Res..

[21] Otsuka M., Ohfusa T., Matsuda Y. (2000). Effect of the binders on polymorphic transformation kietics of carbamazepine in aqueous solution. Colloids Surf. B Biointerfaces.

[22] Raghavan S.L., Trividic A., Davis A.F., Hadgraft J. (2001). Crystallization of hydrocortisone acetate: Influence of polymers. Int. J. Pharm..

[23] Aaltonen J., Heinanen P., Peltonen L., Kortejarvi H., Tanninen V.P., Christiansen L., Hirvonen J., Yliruusi J., Rantanen J. (2006). In situ measurement of solvent-mediated phase transformations during dissolution testing. J. Pharm. Sci..

[24] Karim S., Baie S.H., Hay Y.K., Bukhari N.I. (2014). Development and evaluation of omeprazole pellets fabricated by sieving-spheronisation and extrusion—Spheronisation process. Pak. J. Pharm. Sci..

[25] Kleinebudde P. (1993). Application of low substituted hydroxypropylcellulose (L-HPC) in the production of pellets using extrusion/spheronisation. Int. J. Pharm..

[26] Majidi S., Motlagh G.H., Bahramian B., Kaffashi B., Nojoumi S.A., Haririan I. (2013). Rheological evaluation of wet masses for the preparation of pharmaceutical pellets by capillary and rotational rheometers. Pharm. Dev. Technol..

[27] Sousa J.J., Sousa A., Podczeck F., Newton J.M. (2002). Factors influencing the physical characteristics of pellets obtained by extrusion-spheronisation. Int. J. Pharm..

[28] Wu S. (1971). Calculation of interfacial tension in polymer systems. J. Polym. Sci. C Polym.Symp..

